# Cytotoxic T-Cell Trafficking Chemokine Profiles Correlate With Defined Mucosal Microbial Communities in Colorectal Cancer

**DOI:** 10.3389/fimmu.2021.715559

**Published:** 2021-09-01

**Authors:** Jiali Zhang, Ji Tao, Ruo-Nan Gao, Zhi-Yuan Wei, Yu-Shan He, Chun-Yan Ren, Qi-Chun Li, Yan-Shan Liu, Ke-Wei Wang, Gong Yang, Chengjia Qian, Jian-Huan Chen

**Affiliations:** ^1^Laboratory of Genomic and Precision Medicine, Wuxi School of Medicine, Jiangnan University, Wuxi, China; ^2^Central Laboratory, The Fifth People’s Hospital of Shanghai Fudan University, Shanghai, China; ^3^Department of Hospital Infection, Affiliated Hospital of Jiangnan University, Wuxi, China; ^4^Cancer Institute, Fudan University Shanghai Cancer Center, Shanghai, China; ^5^Department of General Surgery, Affiliated Hospital of Jiangnan University, Wuxi, China

**Keywords:** T cell trafficking, mucosa-associated bacteria, tumor, adjacent normal tissues, colorectal cancer, chemokines

## Abstract

The involvement of gut microbiota in T-cell trafficking into tumor tissue of colorectal cancer (CRC) remains to be further elucidated. The current study aimed to evaluate the expression of major cytotoxic T-cell trafficking chemokines (CTTCs) and chemokine-associated microbiota profiles in both tumor and adjacent normal tissues during CRC progression. We analyzed the expression of chemokine C-X-C motif ligands 9, 10, and 11 (*CXCL9*, *CXCL10*, and *CXCL11*), and C-C motif ligand 5 (*CCL5*), characterized gut mucosa-associated microbiota (MAM), and investigated their correlations in CRC patients. Our results showed that the expression of *CXCL9, CXCL10*, and *CXCL11* was significantly higher in tumor than in adjacent normal tissues in 136 CRC patients. Notably, the high expression of *CXCL9* in tumor tissues was associated with enhanced CD8^+^ T-cell infiltration and improved survival. Moreover, the MAM in tumor tissues showed reduction of microbial diversity and increase of oral bacteria. Microbial network analysis identified differences in microbial composition and structure between tumor and adjacent normal tissues. In addition, stronger associations between oral bacteria and other gut microbes were observed. Furthermore, the correlation analysis between the defined MAM and individual CTTCs showed that the CTTCs’ correlated operational taxonomic units (OTUs) in tumor and adjacent normal tissues rarely overlap with each other. Notably, all the enriched OTUs were positively correlated with the CTTCs in either tumor or adjacent normal tissues. Our findings demonstrated stronger interactions between oral bacteria and gut microbes, and a shifted correlation pattern between MAM and major CTTCs in tumor tissues, underlining possible mechanisms of gut microbiota–host interaction in CRC.

## Introduction

Colorectal cancer (CRC) accounts for the third most commonly diagnosed cancer and the second leading cause of cancer-related death worldwide (1). High infiltration with cytotoxic T cells (CTCs) correlates with improved relapse-free and overall survival (OS) in patients with CRC (2–4). Therefore, recent therapeutic strategies for cancer such as immunotherapies focus on CTC trafficking to the tumor site (5–8). Different from the remarkable treatment responses of adoptive immunotherapy in patients affected by advanced melanoma and hematologic malignancy, the effect of immunotherapy against CRCs has been more moderate (9). One of the major challenges is to effectively traffic CTCs to the tumor microenvironment (10, 11).

The expression of chemokine receptors in CTCs, as well as the expression of their ligands in tumor tissues, is essential for localizing CTCs to tumor tissue (5, 12, 13). It has been reported that CTCs with higher CXCR3 expression can be recruited to the tumor site by ligands, including CXCL9, CXCL10, and CXCL11, which are known as IFNγ-inducible chemokines (12, 13). Additionally, studies have identified CCL5 and its receptor (C-C motif) receptor 5 (CCR5) as another critical component of T-cell chemotaxis that is closely associated with CTC infiltration and better survival (12). Therefore, expression of specific chemokines in tumors could be potentially correlated with the presence of CTCs, which might serve as useful targets for anti-cancer therapies.

The gut mucosa is a dynamic interface between host cells and microbiota (14). Progress of colorectal neoplasia has been linked to interactions between tumor microenvironment and mucosal microbiota barrier, whose process can be reversed by interfering the microbiota (15–17). In mouse models, mixture of microbes enhances anti-cancer immunity through inducing interferon-γ-producing CTCs in the tumor tissues (18), pointing to plausible evidence for the use of gut microbiota as a therapeutic target. Additionally, it has recently been demonstrated that bacteria isolated from CRC tissues could upregulate expression of most types of CTTCs in CRC cell lines *in vitro* (19). However, it remains to be elucidated the change of microbiome profiles during the transition from normal mucosae to malignant lesions, and the correlation between CTTCs and defined microbial communities in the CRC tumor microenvironment. Although enrichment of *Fusobacterium* and its regulation of tumor microenvironment have been demonstrated in CRC (20, 21), increasing evidence suggests that microbiota work as a community with nonnegligible contribution from various microbes (22–24), which remains to be clarified. Herein, we investigated the microbial transition in the tumor mucosae and adjacent normal mucosae, and the association between bacterial colonization and CTTCs in CRC patients.

## Materials and Methods

### Patient Recruitment and Sample Collection

A total of 136 CRC patients scheduled for a primary resection of their tumor at the Affiliated Hospital of Jiangnan University between 2016 and 2019 were recruited in the study. The participants did not receive chemo-radiotherapy before the resection and were not treated with antibiotics in the month prior to surgery but were administered antibiotics intravenously within a few hours of the resection. After surgery, there were 136 pairs of fresh tissues from colorectal tumor or as far away from the tumor as possible (adjacent tissue) collected. Biopsies were snap-frozen in a cryovial immediately with liquid nitrogen and then stored at −80°C until DNA extraction. Histopathological and clinical features were scored according to the International Union Against Cancer (UICC)–TNM staging system. This study was approved by the Ethics Committee of Jiangnan University and was conducted in accordance with the Declaration of Helsinki. Informed consent was obtained from all of the participants after explanation of the nature of the study.

### DNA Extraction and 16S rRNA Gene Sequencing

Paired mucosae samples from tumor and adjacent normal tissues were subjected to DNA extraction. Mucosal DNA was extracted using the AllPrep DNA/RNA extraction kit. Total DNA was purified from tumor and paired normal adjacent tissue samples. MAM was analyzed based on 16S rRNA gene sequencing. 16S rRNA gene amplicon sequencing was carried out employing the 16S rRNA gene Sequencing Library Preparation protocol developed by Illumina (San Diego, California, USA). Briefly, 200 ng of mucosal DNA was amplified from each sample using the primers 515F (5′ GTGCCAGCMGCCGCGGTAA 3′) with Titanium Adaptor B and 806R (5′ GGACTACHVGGGTWTCTAAT 3′) with Titanium Adaptor A and a sample-specific barcode sequence targeting the V4 hypervariable region of the 16S rRNA gene using FastStart Taq DNA Polymerase (Roche, USA).

### RNA Isolation, mRNA Expression, and Quantitative PCR

Total RNA was isolated from CRC tumor tissues and paired normal adjacent tissue using Trizol (Invitrogen, USA). The obtained RNA was used to synthesize cDNA by Superscript III Reverse Transcriptase (Promega, USA). Real-time PCR reaction mixes were prepared using SYBR Green (TaKaRa, Japan) and run on the LightCycler^®^ 480 II Real-time PCR System (Roche, USA) with the following conditions: 95°C for 5 min, 95°C for 5 s, and 60°C for 30 s, for 40 cycles. The relative expression level of CTTC mRNA was calculated using the 2^−ΔCt^ (dCt) method. The ΔCt value was calculated by subtracting the Ct value of the housekeeping gene [glyceraldehyde-3-phosphate dehydrogenase (GAPDH)] from that of the target genes. The primers used were as follows: *CXCL9*, forward primer (5′ AAGC AGCCAAGTCGGTTAGT 3′) and reverse primer (5′ CAGCAGTGTGAGCAGTGATTC 3′); *CXCL10*, forward primer (5′ AGCAGAGGAACCTCCAGTCT 3′) and reverse primer (5′ AGGTACTCCTTGAATGCCACT 3′); *CXCL11*, forward primer (5′ GAGTGTGAAGGGCATGGCTA 3′) and reverse primer (5′ CCTTGAACA 3′); *CCL5*, forward primer (5′ CAGTCGTCCACAGGTCAAGG 3′) and reverse primer (5′ CTTGTTCAGCCGGGAGTCAT 3′); *GAPDH*, forward primer (5′ TGACTTCAACAGCGACACCCA 3′) and reverse primer (5′ CACCCTGTTGCTGTAGCCAAA 3′). Experiments were repeated in triplicate.

### Immunohistochemistry and Immunofluorescence Staining and Image Analysis

Specimens used for immunohistochemistry and immuno-fluorescence staining were obtained immediately after surgical resection and fixed in 10% neutral formalin, paraffin-embedded, and used for histological assays as previously described (25). Immunohistochemistry of paraffin sections was carried out using a two-step protocol. Briefly, 5-μm paraffin sections were first deparaffinized and hydrated, and endogenous peroxidase activity was blocked by incubating the slides in 0.3% H_2_O_2_. Antigen retrieval was performed by microwave treatment in citrate buffer, pH 6.0. Sections were blocked with normal sera from the same species from which the secondary antibodies were derived. After overnight incubation at 4°C with antibodies against human CD8 (1:300 dilution, Abcam, ab101500), CXCR3 (1:300 dilution, ABclonal, A2939), or control antibodies (Rabbit monoclonal IgG, Abcam, ab172730), we applied Envision System HRP-labeled polymer anti-rabbit (for CD8 and CXCR3) (Dako Cytomation) for 30 min and diaminobenzidine (5 min) and hematoxylin counterstain (1 min). Slides were scanned by an automated scanning microscope (Pannoramic Digital Slide Scanners, 3DHISTECH). The 3DHISTECH software (CaseViewer) was used to count the number of positive signals in each tissue core. We calculated the average density (cells/mm^2^) of each tumor-infiltrating CD8^+^ T-cell subset or CXCR3^+^ subset.

For multiple-color immunofluorescence staining, formalin-fixed, paraffin-embedded sections were deparaffinized and rehydrated. Antigen retrieval was performed by microwave treatment in citrate buffer, pH 6.0, and blockage of non-specific antibody binding was carried out with 5% BSA. Sections were then incubated with anti-human CXCR3 and CD3 overnight at 4°C, followed by specimen-paired immunofluorescence secondary antibodies. Negative controls were generated by replacing primary antibodies with isotype-matched antibodies. Slides were analyzed on a fluorescent imaging microscope.

### Bioinformatics Analysis

The Quantitative Insights into Microbial Ecology version 2 (QIIME2) software (subversion 2019.1) was used for quality filtering of DNA sequences, demultiplexing, taxonomic assignment, and calculating α- and β-diversity. For details, selected sequences were clustered into OTUs with USEARCH (version 11, http://drive5.com/uparse/), with a threshold sequence identity of 99%. The reads were aligned to the Greengenes Core Set reference alignment using PyNAST. The Greengenes taxonomies were used to generate summaries of the taxonomic distributions of OTUs across different levels (phylum, order, family, genus, and species). A phylogenetic tree was built with FastTree and used for estimates of α-diversity (Rarefaction curves, Chao1, Shannon diversity) and β-diversity (using unweighted UniFrac). Metagenomic content of the microbiota samples was predicted from the 16S rRNA profiles, and KEGG pathway functions were categorized at Levels 1–3 using the phylogenetic investigation of communities by reconstruction of unobserved states (PICRUSt) tool (26).

### Statistical Methods

All data were summarized as means ± SEM and analyzed with SPSS software (Version 22). Comparisons between CTTC groups were performed by Student’s *t*-test. Correlations between continuous variables were determined by Pearson correlation analysis. Survival was estimated by the Kaplan–Meier method and compared by the log-rank test. Multivariate analysis of prognostic factors for OS was performed using the Cox proportional hazards model. The *χ* ^2^ test was used to test for relationships between categorical variables. Values of *p* < 0.05 (two-tailed) were considered significant.

For microbiota, differential abundance analyses were performed using paired Wilcoxon signed-rank test to identify significant changed features between tumor and adjacent normal mucosae. Using the R implementation of Random Forests 10-fold cross-validations with 100 iterations, we selected a minimum set of bacterial taxa that maximally discriminated against each Dukes stage, different patients’ survival state, and lymph node metastasis state; the variable importance of a microbial taxon was determined by 100 iterations of the algorithm with 3,000 trees and the default mtry of p^1/2^, where p is the number of input phylotypes.

## Results

### Expression of CTTCs in CRC Tissues and Their Association With Disease Progression

**Figure 1:**
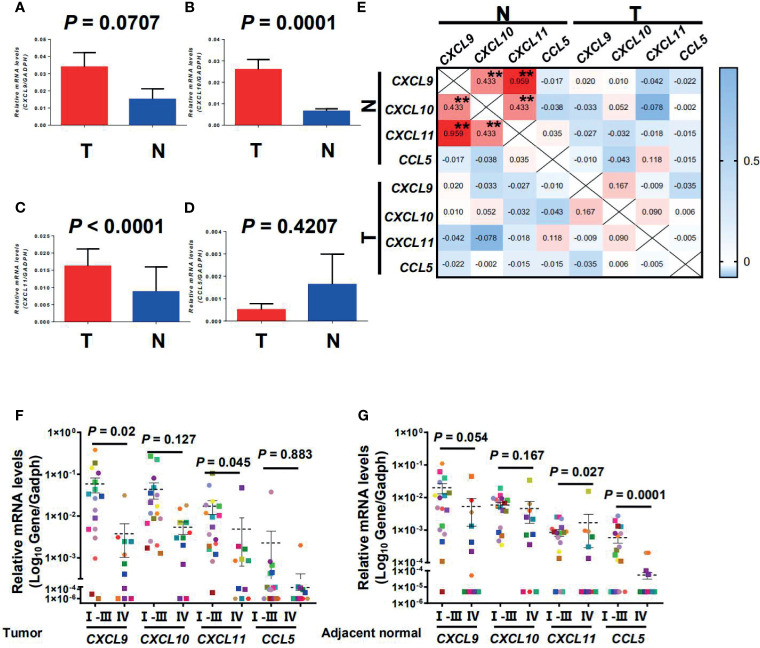
*CXCL9, CXCL10, CXCL11*, and *CCL5* were selectively regulated in tumor and adjacent normal tissues and decreased with progressive stages in CRC patients. Quantitative real-time polymerase chain reaction (qPCR) results of gene expression are shown for *CXCL9, CXCL10, CXCL11*, and *CCL5* in tumor and adjacent normal tissues (*n* = 136) **(A–D)**. T, Tumor; N, Adjacent normal. Results are expressed as means ± SEM. **(E)** Correlation between the relative expression level of CTTCs in tumor and adjacent normal tissues. Pearson correlation coefficients calculated from the relative expression of CTTCs are shown. **p < 0.001. **(F, G)** Representative dot plots of at least three individuals from more than three independent experiments; the continuous and dashed horizontal bars in **(F, G)** represent median values.

CTTC mRNA expression in tumor and adjacent normal tissues from 136 CRC patients was analyzed using quantitative PCR. The expression of *CXCL9*, *CXCL10*, and *CXCL11* except for *CCL5* was significantly higher in the tumor compared with the adjacent normal tissues (**Figures 1A–D**; *p* = 0.0707, *p* = 0.0001, *p* < 0.0001, and *p* = 0.4207). Correlation analysis showed that several chemokines were significantly associated with one another in adjacent normal tissues but not tumor tissues, indicating that different chemokines might be regulated simultaneously in adjacent normal tissues during tumor progression (**Figure 1E**).

In CRC, the expression level of CTTCs in tissues can significantly impact the distribution of CTCs and then the patients’ clinical outcome (2–4). Therefore, we further examined the expression level of CTTCs from patients at different stages of CRC. In tumor, the expression level of chemokine *CXCL9* and *CXCL11* was significantly decreased in advanced stage CRC patients (stages IV, *n* = 11; *p* = 0.02 and *p* = 0.045; **Figure 1F**) compared to those in early stages (stages I, II and III, *n* = 18). Moreover, the expression level of chemokine *CXCL11* and *CCL5* in the adjacent normal tissues was significantly lower in advanced stage CRC (stages IV, *n* = 11; *p* = 0.027 and *p* = 0.0001; **Figure 1G**) than those in early stages (stages I, II, and III, *n* = 18). In addition, no significant difference was observed in the expression level of *CXCL10* in the tumor or adjacent normal tissues between patients in advanced stage and those in early stages (*p* = 0.127 for tumor and *p* = 0.167 for adjacent normal; **Figures 1F, G**). Collectively, the results indicated that the three CTTCs (*CXCL9, CXCL11*, and *CCL5*) were selectively regulated in tumor or adjacent normal tissues and decreased with progressive stages in CRC patients.

### Association Between CTTC Expression and Local CD8^+^ T-Cell Infiltration

**Figure 2:**
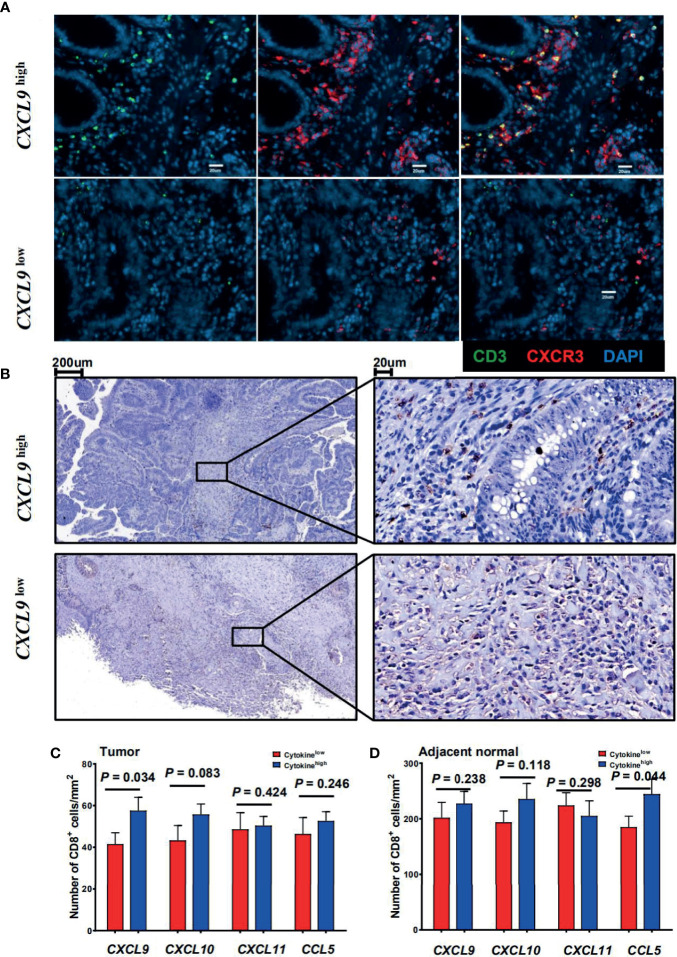
Association between the cytotoxic T-cell trafficking chemokine expression and local CD8^+^ T-cell infiltration. **(A)** Immunofluorescence microscopy of CRC tissue sections from CRC patients with high or low *CXCL9* expression levels, and stained with anti-CD3 (green) and anti-CXCR3 (red) monoclonal antibodies. **(B)** CD8 (brown) immunostaining of tumor tissues from CRC patients. Representative images of low (upper panel) or high (lower panel) CD8 score are shown. **(C, D)** Comparison of the mean ( ± SEM) of CD8^+^ cell densities in high (blue bars) and low (red bars) expression levels of individual chemokines within the tumor (left) or adjacent normal tissues (right) from CRC patients (*n* = 29).

The CRC patients were then categorized into two groups according to the expression level of CTTCs (*CXCL9*, *CXCL10*, *CXCL11*, and *CCL5*) in tumor. To evaluate the potential role of these chemokines in the localization of immune cells, immunofluorescence staining was applied to examine the *in situ* infiltration of CXCR3^+^ and CD3^+^ cells in CRC patients. CXCR3^+^ cells were enriched in tumor tissues from *CXCL9* ^high^ patients, which also was the hotspot for CXCR3^+^ CD3^+^ cells (**Figure 2A**). Additionally, the infiltration density of CD8^+^ T cells in tumor and adjacent normal tissues was assessed and compared between patients with high and low CTTC expression (**Figures 2B–D**). We found that the density of CTCs was significantly higher in tumors in the *CXCL9* ^high^ group than that in the *CXCL9* ^low^ group (**Figure 2C**). In adjacent normal tissues, the density of CTCs was higher in the *CCL5* ^high^ group than in the *CCL5* ^low^ group (**Figure 2D**). Taken together, the results showed that the *CXCL9* ^high^ group in tumor or *CCL5* ^high^ group in adjacent normal tissues exhibited relatively higher CTC infiltration than the matched chemokine low group, implicating altered anti-tumor immune activity.

### Prognostic Significance of CTTCs in CRC Patients

To address whether CTTC expression was associated with CRC progression, we analyzed the correlation between relevant clinical features and the expression level of these chemokines in tumor or adjacent normal tissues from 136 patients. Using the median value (50–50 division), the expression level of *CXCL9, CXCL10, CXCL11*, and *CCL5* in tumor or adjacent normal tissues allowed the stratification of patients into groups. We first determined if any significant associations existed between clinical characteristics and these chemokines. The results showed that in tumor, the expression of *CXCL9* negatively correlated with tumor stage, nodal and distant metastases, and Dukes’ stage (*p* = 0.01, *p* = 0.005, *p* = 0.005; **Table 1**), but not with the other three chemokines (data not shown).

**Table 1:** Association of *CXCL9* mRNA expression levels in tumor with clinicopathologic characteristics.

Variables	No. of patients (%)		*p*
	Low	High	
Age, years			0.583
≤64	22	19	
>64	29	30	
Gender			0.509
Male	32	28	
Female	19	21	
Tumor (T) stage			0.494
pTis + pT1 + pT2 + pT3	16	9	
pT4	35	40	
Nodal (N) status			**0.010**
Negative	18	28	
Positive	33	21	
Distant metastases (M)			**0.005**
Negative	43	49	
Positive	7	1	
Dukes’ stage			**0.005**
A + B	17	44	
C + D	34	5	
Differentiation			0.894
Low	32	30	
High + Moderate	19	19	
Vascular invasion			0.465
Absent	12	9	
Present	39	40	
MSI status			0.494
MSS/MSI-low	48	45	
MSI-high	3	4	
Location			0.622
Colon	38	26	
Rectal	13	23	

The bold face indicates p-values of variables with significance in either univariate or multivariate analyses.

**Figure 3:**
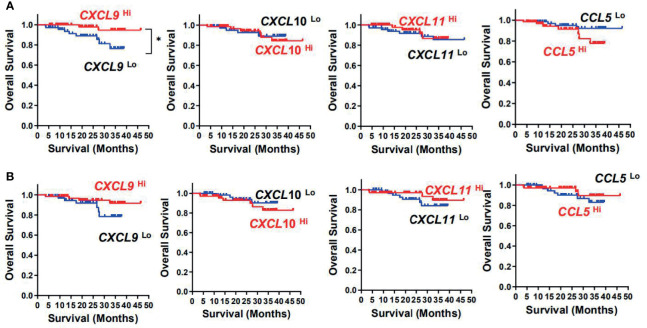
Prognostic significance of *CXCL9, CXCL10, CXCL11*, and *CCL5* in CRC patients. Cumulative OS times were calculated by the Kaplan–Meier method and analyzed by the log-rank test. The patients were divided into two groups according to the median value of *CXCL9, CXCL10, CXCL11*, and *CCL5* in tumor **(A)** or adjacent normal tissues **(B)**.

Kaplan–Meier survival curves further showed positive correlations between *CXCL9* expression level and OS (*p* = 0.048) and disease-free survival (DFS) (*p* = 0.032) in tumor tissues, but not with the other three chemokines in tumor or adjacent normal tissues (**Figures 3A, B** and **Table 2**). Multivariate Cox proportional hazards analysis was then performed, with variables associated with survival in univariate analysis adopted as covariates. In multivariate analysis shown in **Table 2**, the *CXCL9* expression level in tumor could not emerge as an independent prognostic factor of both OS (HR, 0.205; 95% CI, 0.043–0.988; *p* = 0.062) and DFS (HR, 0.443; 95% CI, 0.265–0.742; *p* = 0.065). These results suggested that chemokine *CXCL9* was significantly associated with CRC progression, but might not serve as a powerful predictor of CRC survival alone.

**Table 2:** Univariate and multivariate analyses of factors associated with survival and recurrence.

Variables	OS				DFS			
	Univariate *p*	Multivariate			Univariate *p*	Multivariate		
		HR	95% CI	*p*		HR	95% CI	*p*
Age, years (>58/≤58)	0.764			NA	0.971			NA
Gender (female/male)	0.724			NA	0.601			NA
Tumor stage (pT4/pTis + pT1 + pT2 + pT3)	0.133			NA	0.114			NA
Nodal status (pN1 + pN2/pN0)	0.997			NA	0.997			NA
Distant metastases (Pos/Neg)	**0.002**	9.633	2.269–40.895	**0.001**	**<0.0001**	14.048	1.489–6.933	**<0.0001**
Differentiation(H + M/L)	0.535			NA	0.086			NA
*CXCL9*_high_/*CXCL9*_low_ tumor	**0.048**	0.205	0.043–.988	**0.062**	**0.032**	0.443	0.265–.742	**0.065**
*CXCL10*_high_/*CXCL10*_low_ tumor	0.715			NA	0.957			NA
*CXCL*11_high_/*CXCL11*_low_ tumor	0.377			NA	0.256			NA
*CCL5*_high_/*CCL5*_low_ tumor	0.326			NA	0.957			NA
*CXCL9*_high_/*CXCL9*_low_ peri-tumor	0.377	0.205	0.043–.988	NA	0.092			NA
*CXCL10*_high_/*CXCL10*_low_ peri-tumor	0.326			NA	0.957			NA
*CXCL11*_high_/*CXCL11*_low_ peri-tumor	0.377			NA	0.256			NA
*CCL5*_high_/*CCL5*_low_ peri-tumor	0.357			NA	0.092			NA
Location (rectal/colon)	0.564			NA	0.630			NA

Cox proportional hazards regression model; variables associated with survival by univariate analysis were adopted as covariates in multivariate analyses.

OS, overall survival; DFS, disease-free survival; HR, hazard ratio; CI, confidence interval; NA, not applicable; Pos, positive; Neg, negative.

The bold face indicates p-values of variables with significance in either univariate or multivariate analyses.

### Difference of MAM Between Tumor and Adjacent Normal Mucosae

**Figure 4:**
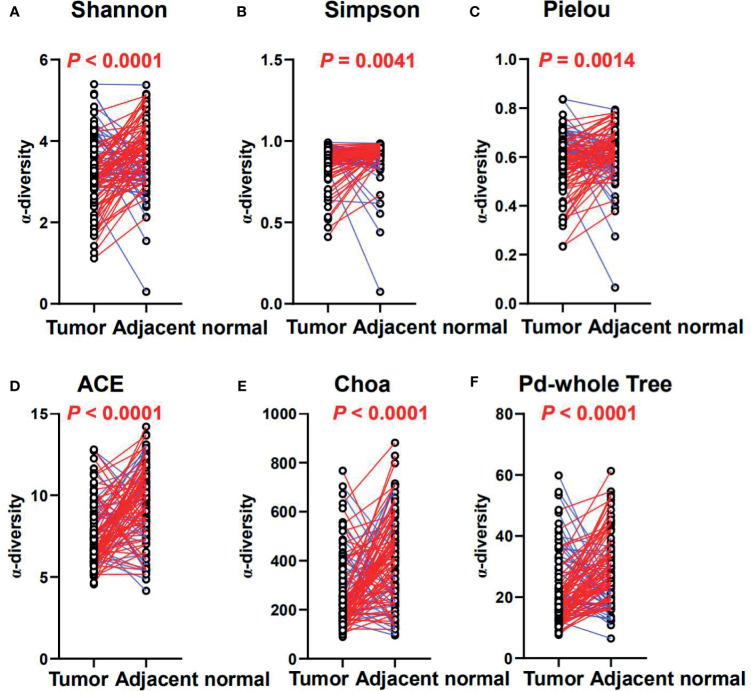
The diversity and richness of the MAM in tumor and adjacent normal mucosae. The diversity indices, such as Shannon **(A)**, Simpson **(B)**, and Heip evenness **(C)**, and the richness indices, such as ACE **(D)**, Choa **(E)**, and PD whole tree **(F)**, are used to evaluate the overall structure of the mucosae microbiota in the CRC patients. *p*-values are derived from two-sided Wilcoxon signed rank tests. The colors indicate increase (blue) or reduction (red) of the diversity indices in tumor compared with adjacent normal tissues.

As gut MAM could serve as stimulation that impacted the chemokine expression, we next continued to identify MAM in tumors and paired adjacent normal mucosae from the CRC patients. To investigate how MAM changed in tumors compared with adjacent normal tissues, we compared paired tumor and adjacent normal tissues from 101 patients in our CRC cohort (**Table 3**). Wilcoxon signed-rank tests showed that alpha-diversity indices, including the Shannon and Simpson indices and Pielou’s evenness, were significantly decreased in tumors (*p* < 0.0001, *p* = 0.0041 and *p* = 0.0014, respectively, **Figures 4A–C**), while richness indices, such as ACE, Chao, and phylogenetic diversity (PD) whole tree, were also decreased in tumors (*p* < 0.0001, *p* < 0.0001, and *p* < 0.0001, **Figures 4D–F**) compared with paired adjacent normal tissues. As for beta-diversity, principal coordinate analysis (PCoA) could not separate the microbiomes from tumor and adjacent normal mucosae into different clusters, which could be due to significant inter-individual variation (data not shown).

**Table 3:** Characteristics of Patients.

Patient characteristics	Group 1	Group 2	Group 3
No. of patients	136	29	101
Age, years (median, range)	64, 21–88	64, 29–79	64, 21–88
Gender (male/female)	81/55	19/10	58/43
Tumor (T) stage (pTis + pT1 +pT2 + pT3/pT4)	43/93	19/10	28/73
Nodal (N) status (Negative/Positive/Nx)	61/74/1	18/11	46/54/1
Distant metastases (M) (None detected/Present)	125/11	18/11	94/7
Dukes’ stage (A + B/C + D)	60/76	17/12	46/55
No. of lymph nodes analyzed (median, range)	10, 1–27	14, 4–26	15, 0–40
Differentiation (Well/Moderate/Poor)	3/81/52	3/15/11	2/56/43
MSI status (MSS+MSI-low/MSI-high)	126/10	27/2	95/6
Location (right side of colon/transverse colon/left side of colon/sigmoid colon/rectum)	15/12/35/26/48	6/1/4/5/13	27/10/15/16/33
Mucinous (colloid) adenocarcinoma (No/Yes)	0/136	0/29	0/101

Nx, Nodal status cannot be assessed in one patient.

Group 2 patients are included in immunohistochemistry and immunofluorescence analysis.

Group 3 patients are included in gut microbiota analysis.

Both Group 2 and Group 3 are included in Group 1, the total patient group.

**Figure 5:**
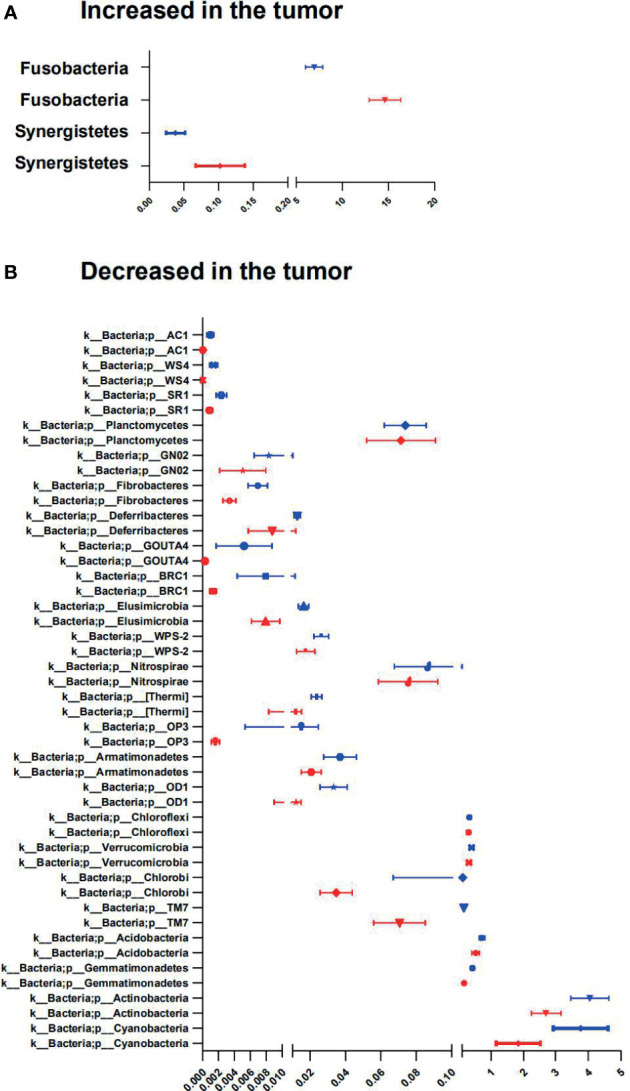
Phyla involved in tumor-associated signature with CRC. **(A, B)** Relative abundance of significantly different phyla between tumor and adjacent normal tissues. The two-sided Wilcoxon signed rank test was used to evaluate the importance of comparisons. Tumor (red bars), Adjacent normal (blue bars).

In our CRC cohort, abundance changes of operational taxonomic units (OTUs) were found at multiple taxonomic levels between the tumor and paired adjacent normal mucosae (**Figures 5A, B**; **Supplementary Figure S2** and **Additional Files 1–6**). At the phylum level, significant changes were found between the tumor and paired adjacent normal mucosae [false discovery rate (FDR) *q* ≤ 0.1], with enrichment of Fusobacteria and Synergistetes, and decrease of Cyanobacteria, Actinobacteria, Gemmatimonadetes, Acidobacteria, TM7, Chlorobi, Verrucomicrobia, Chloroflexi, OD1, Armatimonadetes, OP3, [Thermi], Nitrospirae, WPS-2, Elusimicrobia, BRC1, GOUTA4, Deferribacteres, Fibrobacteres, GN02, Planctomycetes, SR1, WS4, and AC1 in the tumor mucosae (**Figures 5A, B** and **Additional File 6**). In general, 225 gut microbiota OTUs (grouped at 99% sequence identity) were differentially abundant between tumor and adjacent normal mucosae (FDR *q* ≤ 0.1; **Additional File 1**). Most of the differentially abundant OTUs (19/225) were less abundant in the tumor than in the adjacent normal mucosae. Of note, taxa that were detected in over 90% of the patients and were enriched in tumors included *Fusobacterium*, *Bacteroides*, *Stenotrophomonas*, *Lactobacillus*, and *Parvimonas* genera (**Supplementary Figures S2A–D**). The widely distributed tumor microbiomes showed decreased abundances of taxa within the order *Streptophyta* and *Bacteroidales*, also the family *S24-7* and *Rikenellaceae*, as well as species, namely, *Parabacteroides distasonis, Bacteroides uniformis, Akkermansia muciniphila*, and *Clostridium ramosum*, and species in *Turicibacter* genus (**Figures S2E–J** and **Additional File 1**).

### Large Centralities of Oral Bacteria in CRC MAM Network

**Figure 6:**
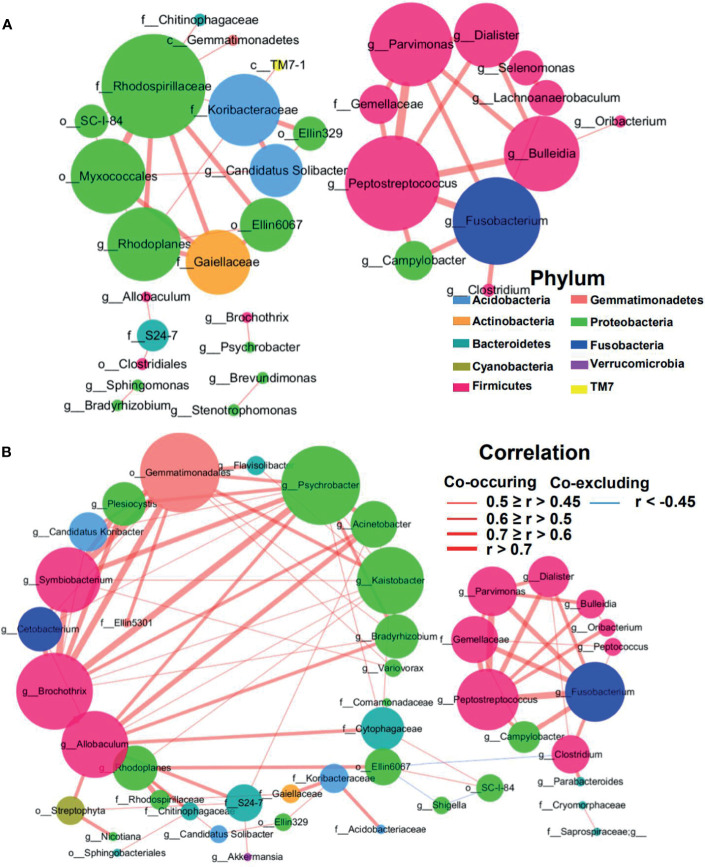
Correlation network of differential MAM in tumor **(A)** and adjacent normal mucosae **(B)**. The correlation coefficients were calculated with the Sparse Correlations for Compositional data algorithm (SparCC). A subset of significant correlations with strengths of at least 0.45 in the mucosae commensals at the genera level were selected for visualization using Cytoscape version 3.6. The size of the nodes corresponds to weighted node connectivity (WNC) scores. Red and green lines represent positive and negative correlations, respectively.

The structure change of the mucosae microbiota is the result of dynamic interactions between community members. The SparCC algorithm was employed to construct microbial interaction networks. Here, we observed both co-occurrence and co-excluding interactions of significantly different OTUs between the tumor and adjacent normal mucosae (**Figures 6A, B** and **Supplementary Figures S3A, B**). As shown in **Figure 6A**, the microbial network interactions in tumors mainly occurred among genera belonging to phyla Firmicutes and Proteobacteria. Notably, co-occurrence interactions among oral bacteria *Fusobacterium*, *Peptostreptococcus*, *Parvimonas*, *Gemellaceae*, and *Campylobacter*, and between *Fusobacterium* and *Clostridium* were observed in both tumor and adjacent normal network. In addition, co-occurrence interactions between oral bacteria *Fusobacterium* and *Lachnoanaerobaculum*, between *Fusobacterium* and *Bulleidia*, and between *Lachnoanaerobaculum* and *Selenomonas* were exclusively observed in tumors. The mucosae microbiota in adjacent normal tissues was also dominated by Firmicutes and Proteobacteria and consisted mainly of the six phyla observed in the tumor group (**Figure 6B**). However, the richer interaction network incorporated more commensal bacteria, with interactions among the family *S24-7, Streptophyta*, and *Acinetobacter* presented in adjacent normal tissues.

Weighted node connectivity (WNC) scores were calculated to determine hub microbes with significant roles in CRC microbial ecological network. To prioritize differentially abundant taxa, we next focused on OTUs with large WNC scores, which indicated large centralities and importance in the CRC MAM interaction network. These taxa with large centralities included genera *Peptostreptococcus, Fusobacterium, Parvimonas, Bulleidia, Rhodoplanes, Candidatus Solibacter, Dialister, Lachnoanaerobaculum*, and *Selenomonas* in tumors (**Figure 6A**). Species-level identification of these OTUs included *Clostridium aldenense, Lachnoanaerobaculum orale, Bacteroides fragilis, Ruminococcus bromii, Bacteroides uniformis*, and *Parabacteroides distasonis* (**Supplementary Figure S3A**). Their centralities suggested that they can form a backbone of niche-specific relationships and might exhibit significant influence on the tumor microbial ecology.

### Altered MAM Functions in Tumors

To determine the potential functional impact of taxonomic changes in CRC MAM, microbial functions associated with CRC in MAM were then predicted by using Phylogenetic Investigation of Communities by Reconstruction of Unobserved States (PICRUSt). Results from Kyoto Encyclopedia of Genes and Genomes (KEGG) pathways showed 18 differentially altered functions at the L2 level between tumor and adjacent normal mucosae with a threshold of Benjamini-Hochberg *p*-values <0.05 (**Supplementary Figure S4**). Functions enriched in adjacent normal tissues compared to tumors at the L3 level such as pathogenic *Escherichia coli* infection, Fc gamma R-mediated phagocytosis, and p53 signaling pathway (**Supplementary Figure S5**). In contrast, 18 pathways at the L3 level were enriched in tumors compared to adjacent normal tissues, such as MAPK signaling pathway, Fc epsilon RI signaling pathway, Carbohydrate digestion and absorption, Lipopolysaccharide biosynthesis, Nucleotide metabolism, D-Alanine metabolism, Epithelial cell signaling in *Helicobacter pylori* infection, Bacterial toxins, Amino acid metabolism, and Antigen processing and presentation.

### Distinctive CTTC Expression Profiles Were Associated With MAM

**Figure 7:**
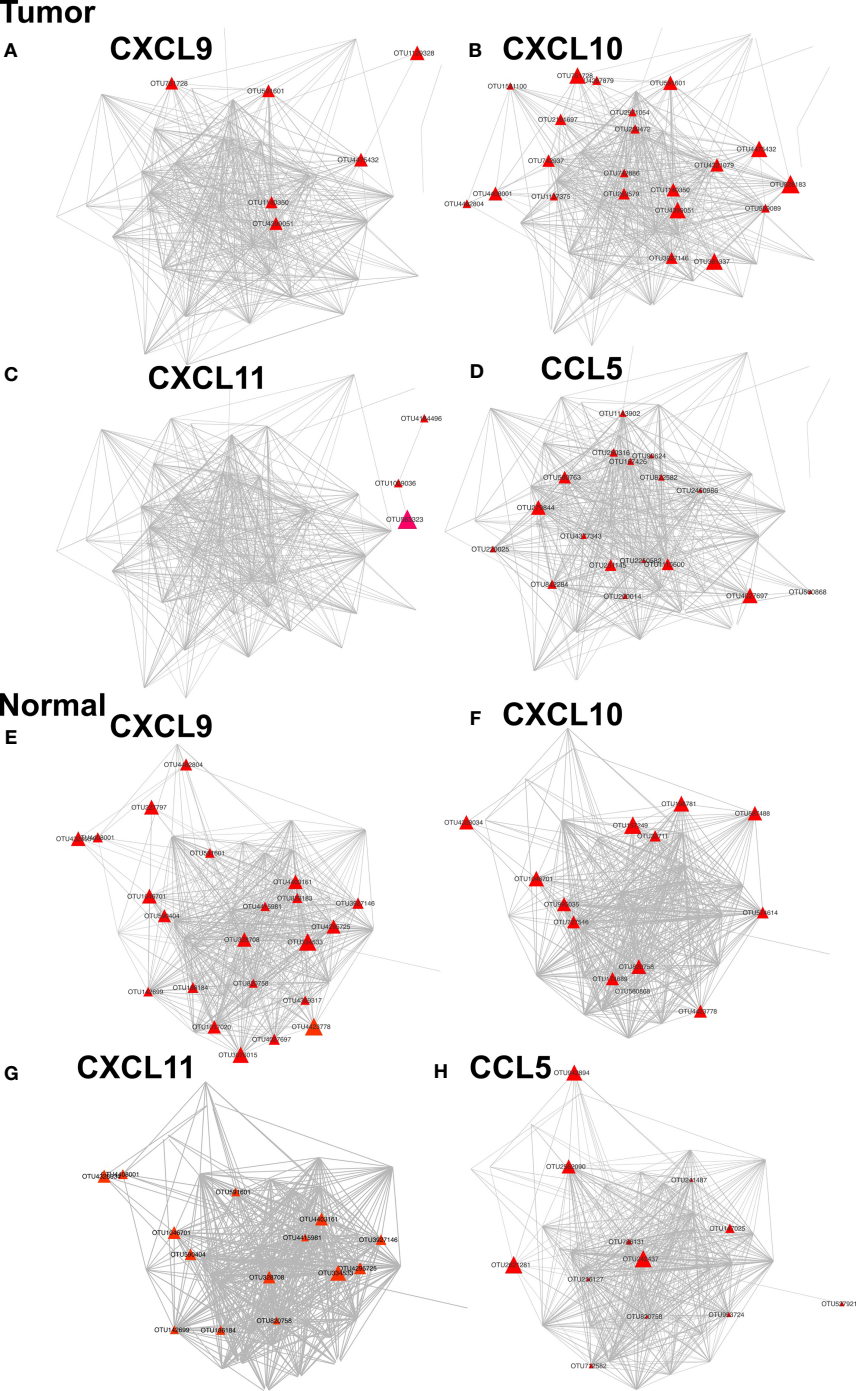
Networks of OTUs associated with CTTCs in tumor and adjacent normal tissues. Each node represents an OTU. Nodes (OTUs) are shown if the abundance of the OTU is significantly correlated with the expression of *CXCL9*, *CXCL10*, *CXCL11*, or *CCL5* in either tumors **(A–D)** or adjacent normal mucosae **(E–H)** (*p* < 0.05). The color and size of nodes denote the *P* value and correlation coefficients (*r*) of correlation between OTUs and CTTCs.

To assess the correlations between the relative abundance of defined MAM and the expression of individual CTTCs in CRC, correlation was analyzed between the expression of CTTCs and the abundance of differently enriched OTUs in tumor compared to adjacent normal mucosal tissues. CTTCs including *CXCL9, CXCL10, CXCL11*, and *CCL5*, which were differentially expressed between tumor and adjacent normal tissues (**Figures 1A–D**), were significantly correlated with the abundance of several OTUs (**Figures 7A–H** and **Additional File 7**). In particular, abundance of several OTUs, mainly including *Acinetobacter lwoffii*, species in *Wautersiella* genus, and *Desulfobacteraceae* family, was significantly correlated with expression of at least two kinds of CTTCs in tumor tissues. It has been reported that *Methylobacteriaceae* was associated with all prognostically favorable T-cell markers and most corresponding recruiting chemokines (19). In line with this, *Methylobacterium adhaesivum* was associated with the high expression of *CXCL10* in tumor tissues. Moreover, *Streptomyces mirabilis*, *Psychrobacter marincola*, *Acinetobacter johnsonii*, *Psychrobacter sanguinis*, and species in *Photobacterium* and *Acinetobacter* genus and *SHA-31* family were significantly correlated with expression of at least two kinds of CTTCs in adjacent normal tissues. Although for each of the CTTCs, their correlated OTUs in tumor and adjacent normal tissues rarely overlap with each other, we still found several OTUs that were correlated with CTTCs in both tumor and adjacent tissues such as *Desulfosporosinus meridiei*, *Rothia mucilaginosa*, and *Haemophilus parasuis*, and species in *B-42* and *Catonella* genus, *Neisseriaceae* and *ML1228J-1* family, and *Spirobacillales* and *GN03* order (**Figures 7A–H**). Notably, taxa that have been reported to be associated with CRC progression (22), such as species in *Fusobacterium* and *Parvimonas* genus, were only positively correlated with the CTTCs in tumor (**Figures 7A–D**). Notably, taxa that have been reported to be associated with CRC progression (22), such as species in *Fusobacterium* and *Parvimonas* genus, were only positively correlated with the CTTCs in tumors (**Figures 7A–D**).

We then analyzed how the MAM was altered during CRC progression. Changes in the microbial communities were identified by comparing the patients at low Dukes’ stages (stages I–II) with those at high Dukes’ stages (stages III–IV) and linear discriminant analysis (LDA) effect size (LEfSe) to compare the taxonomic abundance. In addition, we added the chemokine levels into the risk index to generate the ROC curves (**Supplementary Figures S6A–D**). Our results showed that the changes in abundances of specific taxa in tumor or adjacent normal mucosae can be used as a classifier that distinguishes between low/high Dukes’ stage at a fixed specificity of 81.1% and 81.4% (**Addition File 7**). Besides, the addition of CTTC levels into the risk index would slightly improve the specificity at 83.0% and 88.3%.

## Discussion

In the present study, we aimed to explore the chemotactic factors associated with CRC prognosis and to profile MAM of CRC patients and to investigate the relationship between mucosae microbiota and chemokines involved in CTC recruitment during CRC progression. Based on existing reported clinical data and evidence, we explored major chemokines (including *CCL5*, *CXCL9*, *CXCL10*, and *CXCL11*) associated with recruitment of CTCs into CRC tissues (5, 12, 27–29).

In our current cohort, significant positive correlations between expression of *CXCL9*, *CXCL10*, and *CXCL11* were detected in adjacent normal tissues, suggesting that these chemokines, targeting the same chemokine receptor, may be concomitantly induced and regulated, pointing to their co-expression in normal tissues. Notably, such co-expression was dramatically weakened in tumors, indicating dysregulation of the CTTCs in the tumor microenvironment. Particularly, we found that in tumors, the expression of *CXCL9* negatively correlated with tumor stage, nodal and distant metastases, and Dukes’ stage, and predictive of favorable clinical outcome. Due to the limitation of follow-up time, we could only evaluate the impact on identified chemokines on patients’ 3-year survival instead of 5-year survival in our cohort. Therefore, more follow-up work needs to be completed in the future in order to assess the prognostic value of these CTTCs on the long-term survival and DFS of patients. Previous reports showed that the positive prognostic significance of these chemokines could rely on their capacity to attract the T-cell populations with receptors into tumor tissues (5, 12, 13). In consistent with previous findings, our data also showed that tumors with high *CXCL9* expression level or adjacent normal tissues with high *CCL5* expression level were infiltrated with a higher number of CTCs. It should be noted that a higher-level infiltration of CD8+ T cells does not directly confirm a higher immune activity before further functional validation. Nevertheless, patients with better prognosis outcome might benefit from higher levels of CTTCs within CRC tissues, and more CTC infiltration could possibly be linked to tumor suppression. Recently, Yu et al. observed an increased exhaustion phenotype of intratumoral CD8^+^ T cells that were hyper stimulated by several bacterial species to promote chronic inflammation and consequently tumor development (30), indicating that certain bacterial species could be useful microenvironmental stimuli and dynamically regulate CD8^+^ T-cell function. These findings, taken together, underscore the possibility of CD8^+^ T cells’ functional recovery by gut bacteria intervention, as well as the importance of potential mechanisms on how the microbiome may alter anti-tumor response by CD8^+^ T cells *via* the chemokine–chemokine receptor axis.

Our CRC patients showed heterogeneous CTTC expression and its dysregulation in tumors compared to adjacent normal tissues. Such changes might reflect distinct genetic backgrounds and/or exposure to different microorganisms in the tumor microenvironment. Recent studies reported that stimulation by gut commensal bacteria induced upregulation or *de novo* expression of multiple chemokines in tumor cell lines (18, 19). However, specific microbes in MAM correlated to high chemokine expression, and immune cell infiltration in human CRC samples was not evaluated by far. In the current study, the gut MAM in tumor and adjacent normal tissues showed distinct taxonomic composition, with decreased diversity and richness in tumor tissues. Our findings in CRC are thus in line with a previous hypothesis that altered microbial diversity can be recognized as a feature of disease status, including inflammatory diseases and cancer (31–33).

In addition to confirmation of previously reported association between microbes and CRC, our MAM analysis also revealed novel taxonomic changes in the disease. At the phylum level, *Fusobacteria* was substantially enriched in tumor MAM in our CRC patients, in consistent with previous reports (34–37). Notably, *Synergistetes* could be a novel phylum significantly enriched in tumors, although its abundance was much lower compared with that of *Fusobacteria* in the CRC patients. *Synergistetes* has not previously been reported as a tumor-enriched phylum in gut microbiome studies with European and American CRC cohorts (22, 22, 35), suggesting that this phylum might be a characteristic in our local Chinese CRC cohort. At the genus level, *Fusobacterium* has been reported as the most abundant genus, which resides in the oral cavity as commensals, but can be an opportunistic pathogen for colon carcinogenesis *via* alterations in signaling pathways or impairment of antitumor immune functions (20). In addition, it has been demonstrated that *Fusobacterium nucleatum* plays a role in the development and progression of colon cancer (20, 38–40), and it has also been detected in patient samples with nodal and distant metastasis (41–44). In our current study, we also confirmed a significantly increased abundance of *Fusobacterium* in tumor compared with adjacent normal tissues and in advanced stage CRC patients compared with early-stage CRC patients, suggesting it as an important and dominant candidate pathogen in both occurrence and development of CRC. Moreover, *B. fragilis* was shown in our data to be detected in 95% of CRC tumor tissues, and it could possibly stimulate an inflammatory status that can promote carcinogenesis *via* induction of proinflammatory toxins as reported (45).

It is noteworthy that our findings demonstrate substantial alteration of MAM microbial functions in CRC patients. Pathways related to genetic information processing and bacterial toxin biosynthesis were also found at higher abundance in tumor, which was in line with the increase of bacteria that could synthesize proinflammatory toxins, such as *B. fragilis*. In contrast, microbial pathways associated with metabolism were downregulated in tumor. Such findings, in line with a previous report in other cohorts (22), indicate that during CRC progression, altered microbiome could be involved in shifted host immune response and metabolism, which were key components of carcinogenesis and the maintenance of local microenvironment of CRC.

Previous reports showed that CTTCs might predict favorable outcome in CRC patients (3, 4). However, our data showed that CTTCs could not serve as a powerful predictor of patient survival. Moreover, our results revealed that a set of defined microbes positively associated with the expression of CTTCs in CRC patients, suggesting that intervention of gut microbiota might be useful in targeting the balance of the tumor microenvironment of CRC. Notably, a group of defined microbes in tumor and adjacent normal mucosae were associated with expression of CTTC genes, possibly indicating their capacity to promote recruitment of CTC populations. Most of the correlations between OTUs and CTTCs in tumor and adjacent normal tissues were not found in the other tissue types, in line with the dysregulation of CTTCs in tumors. Interestingly, *Methylobacteriaceae* was positively correlated with *CXCL10* in both tumor and adjacent normal mucosa in our CRC patients, and it has been reported to be associated with prognostically favorable T-cell markers and most corresponding recruiting chemokines (19). The results suggested that in view of the complex tumor microenvironment, both cancer-promoting and cancer-suppressing factors could co-exist in MAM. Considering that species in *Fusobacterium* genus was one of the mucosal bacteria that promoted tumor development, this could partially explain why the chemokine *CXCL9* could not serve as a powerful independent predictor of our CRC patient survival. Importantly, among the microbes positively associated with CTTCs such as *CXCL9*, there were a set of gram-negative bacteria. The enriched gram-negative bacteria in tumors could possibly stimulate an inflammatory state, induce the increase of CTTCs in the tumor microenvironment, and affect the progression and prognosis of CRC patients. Such findings might improve our understanding of the microbiota dynamics along CRC progression and provide new insight into the development of treatment strategies with immune therapy. Further studies are thus warranted to clarify the species-level or strain-level impact of MAM microbes on chemokine secretion in tumor tissues and T-cell infiltration.

In conclusion, the current study reveals the significant correlation between cytotoxic trafficking chemokines and gut MAM in CRC patients and improved prognosis. This knowledge might eventually pave the way towards development of innovative treatments by modifying gut microbiota to promote cytotoxic T-cell infiltration for the favorable prognostic significance.

## Data Availability Statement

Our data has been successfully deposited to NCBI. The accession number is: PRJNA669258.

## Ethics Statement

The studies involving human participants were reviewed and approved by Medical Ethics Committee of Jiangnan University. The ethics committee waived the requirement of written informed consent for participation.

## Author Contributions

JZ and J-HC designed the study, analyzed the data, and wrote the paper. CQ and R-NG developed the clinical sample cohorts, and JT, C-YR, R-NG, and Q-CL managed collection of clinical samples and information, DNA extraction, and quantification. JZ analyzed the NGS data. JZ and K-WW performed the statistical analysis. GY, Y-SL, and J-HC, in addition to all other co-authors, reviewed the manuscript, provided feedback, and approved the manuscript in its final form. All authors contributed to the article and approved the submitted version.

## Funding

This study was supported in part by grants from the National Natural Science Foundation of China (No. 31671311), the Chinese Postdoctoral Science Foundation (No. 2019M651713), Jiangsu Planned Projects for Postdoctoral Research Funds (No. 2018K239C), the Youth Foundation of Jiangsu Natural Science Foundation (No. BK20180617), Guangdong Basic and Applied Basic Research Foundation (No. 2019A1515012062), the Fundamental Research Funds for the Central Universities (JUSRP51712B and JUSRP1901XNC), the Program for High-Level Entrepreneurial and Innovative Talents Introduction of Jiangsu Province, Guangdong High-level Personnel of Special Support Program, Yangfan Plan of Talents Recruitment Grant, the Taihu Lake Talent Plan, Research Program of Public Health Research Center, Jiangnan University (JUPH201827), Youth Research Program of Wuxi Health Commission (Q202011) and Wuxi Institute of Translational Medicine.

## Conflict of Interest

The authors declare that the research was conducted in the absence of any commercial or financial relationships that could be construed as a potential conflict of interest.

## Publisher’s Note

All claims expressed in this article are solely those of the authors and do not necessarily represent those of their affiliated organizations, or those of the publisher, the editors and the reviewers. Any product that may be evaluated in this article, or claim that may be made by its manufacturer, is not guaranteed or endorsed by the publisher.

## Supplementary Material

Supplementary Figure 1 Differential enrichment of representative species between tumor and adjacent normal mucosae microbiomes. Representative dot plots of each tumor tissue with corresponding paired adjacent normal tissue indicating the relative abundances of representative microbes that are presented in over 90% of the patients and are differentially abundant between tumor and adjacent tissues **(A–N)**. The continuous and dashed horizontal bars denotes median abundance. Results are expressed as means ± SEM.

Supplementary Figure 2 Multiple taxonomic levels involved in tumor associated-signature with CRC. **(A–J)** Relative abundance of significantly different taxa between tumor and adjacent normal tissues. The two-sided Wilcoxon signed rank test is used to evaluate the importance of comparisons. Tumor (Red Bars), Adjacent normal (Blue Bars).

Supplementary Figure 3 Correlation network of differential MAM in tumor **(A)**, adjacent normal mucosae **(B)**. The correlation coefficients are calculated with the Sparse Correlations for Compositional data algorithm (SparCC). A subset of significant correlations with strengths of at least 0.4 in the mucosae commensals at the OTU level are selected for visualization. The size of the nodes corresponds to weighted node connectivity (WNC) scores. Cytoscape version 3.6.1 is used for network construction. Red and green lines represent positive and negative correlations, respectively.

Supplementary Figure 4 Representative KEGG pathways **(A)** and level 2 COG **(B)** of MAM that are enriched in tumor or adjacent normal mucosae. PiCRUSt-based CRC MAM functions are analyzed in tumor and adjacent normal tissues. The microbial functions between the two groups are compared based on two-sided Welch’s t-test. The Benjamini-Hochberg method was used for multiple testing correction based on the false discovery rate (FDR) by STAMP.

Supplementary Figure 5 Summary cladogram of differentially abundant KEGG (level3) modules imputed in MAM. Node sizes represent the tumor to adjacent normal relative abundance ratios. Clades and nodes are annotated in a clockwise manner. Functional categories at level 1and 2 are distinguished by respective node shapes and lower-case letters. Node colors represent enriched functions in tumor (red) and those in adjacent normal tissues (blue). The letters on annotation, I and D stands for increased and decreased function in tumors, respectively.

Supplementary Figure 6 ROC curves analysis to evaluate the discriminatory potential of mucosal microbial community combined with CCTC levels in Ducks’ stage identification. **(A)** Microbial community at the tumor mucosal classify Low- Ducks’ stage (stages 1–2) from high- Ducks’ stage (stages 3–4). **(B)** Microbial community combined with CCTC at the tumor mucosal classify Low- Ducks’ stage (stages 1–2) from high- Ducks’ stage (stages 3–4). **(C)** Microbial community at the adjacent normal mucosal classify low-Ducks’ stage (stages 1–2) from high-Ducks’ stage (stages 3–4). **(D)** Microbial community combined with CCTC at the adjacent normal mucosal classify low-Ducks’ stage (stages 1–2) from high-Ducks’ stage (stages 3–4).
