# Supplementary figures and images for: Cytotoxic T-Cell Trafficking Chemokine Profiles Correlate With Defined Mucosal Microbial Communities in Colorectal Cancer
Source: Front Immunol. 2021 Sep 1;12:715559. doi: 10.3389/fimmu.2021.715559 (PMC8442671; doi:10.3389/fimmu.2021.715559)

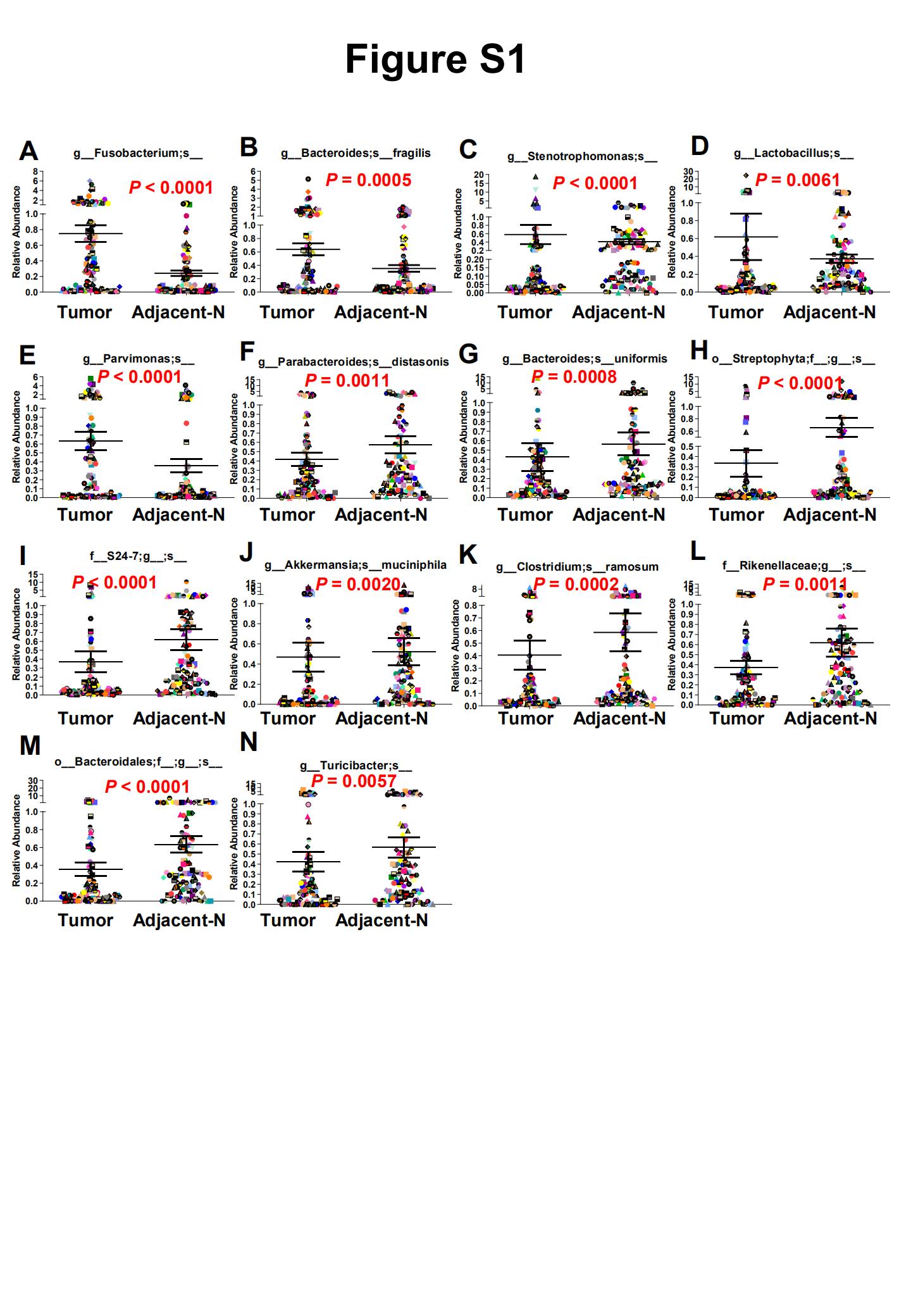

Supplement: Supplementary Figure 1 — Differential enrichment of representative species between tumor and adjacent normal mucosae microbiomes. Representative dot plots of each tumor tissue with corresponding paired adjacent normal tissue indicating the relative abundances of representative microbes that are presented in over 90% of the patients and are differentially abundant between tumor and adjacent tissues (A–N). The continuous and dashed horizontal bars denotes median abundance. Results are expressed as means ± SEM. [file Image_1.jpeg]

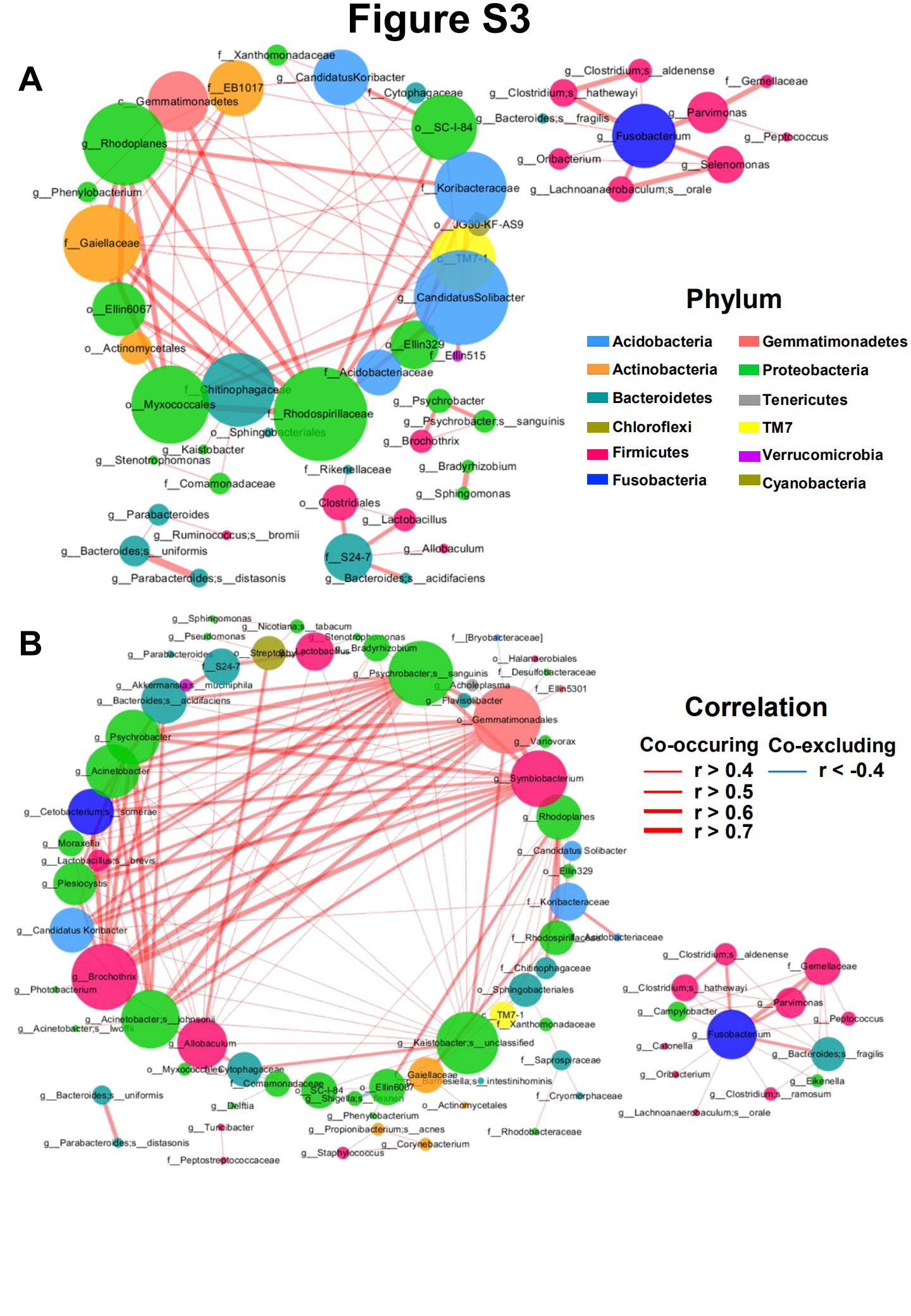

Supplement: Supplementary Figure 3 — Correlation network of differential MAM in tumor (A), adjacent normal mucosae (B). The correlation coefficients are calculated with the Sparse Correlations for Compositional data algorithm (SparCC). A subset of significant correlations with strengths of at least 0.4 in the mucosae commensals at the OTU level are selected for visualization. The size of the nodes corresponds to weighted node connectivity (WNC) scores. Cytoscape version 3.6.1 is used for network construction. Red and green lines represent positive and negative correlations, respectively. [file Image_3.jpg]

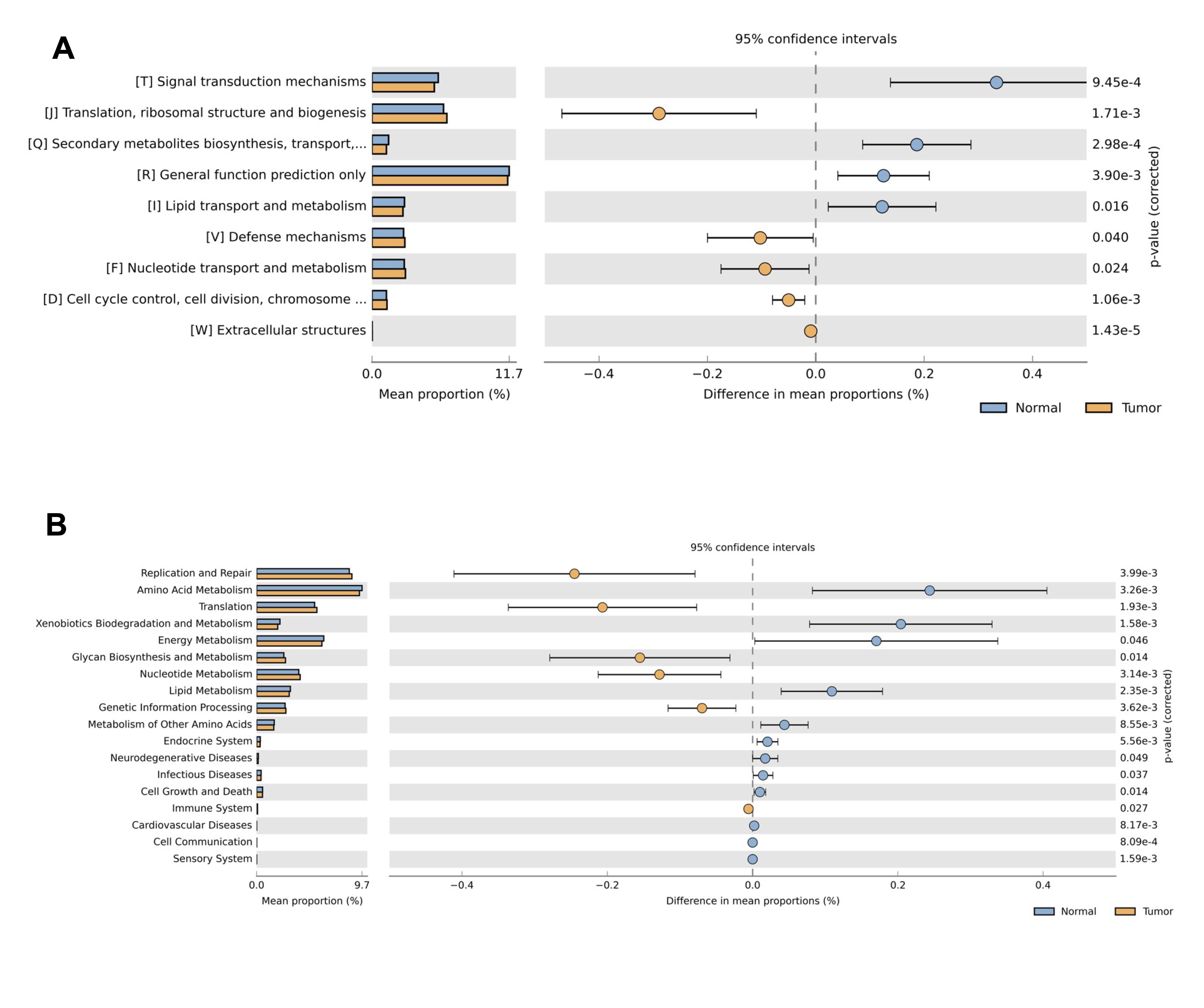

Supplement: Supplementary Figure 4 — Representative KEGG pathways (A) and level 2 COG (B) of MAM that are enriched in tumor or adjacent normal mucosae. PiCRUSt-based CRC MAM functions are analyzed in tumor and adjacent normal tissues. The microbial functions between the two groups are compared based on two-sided Welch’s t-test. The Benjamini-Hochberg method was used for multiple testing correction based on the false discovery rate (FDR) by STAMP. [file Image_4.jpg]

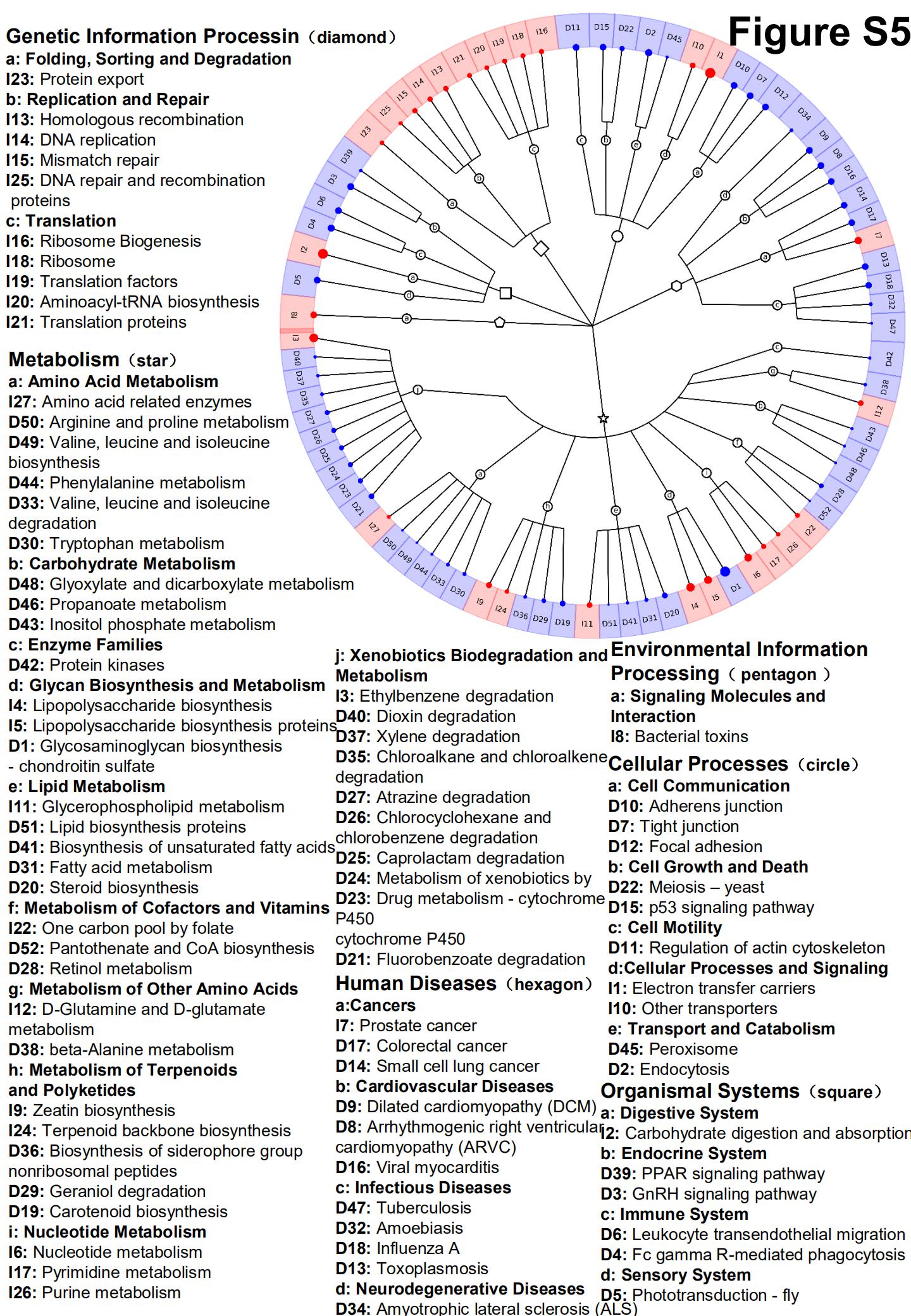

Supplement: Supplementary Figure 5 — Summary cladogram of differentially abundant KEGG (level3) modules imputed in MAM. Node sizes represent the tumor to adjacent normal relative abundance ratios. Clades and nodes are annotated in a clockwise manner. Functional categories at level 1and 2 are distinguished by respective node shapes and lower-case letters. Node colors represent enriched functions in tumor (red) and those in adjacent normal tissues (blue). The letters on annotation, I and D stands for increased and decreased function in tumors, respectively. [file Image_5.jpg]

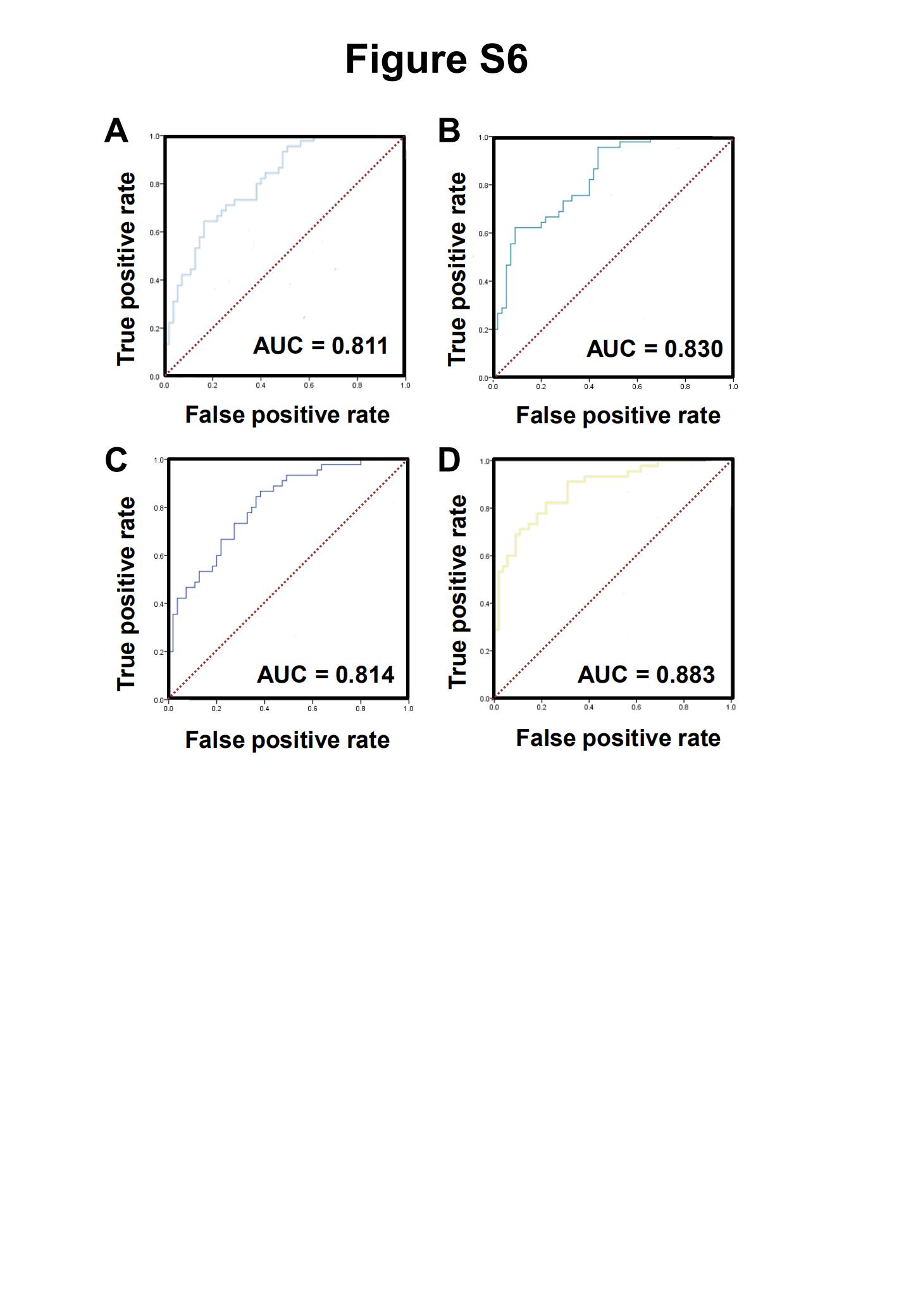

Supplement: Supplementary Figure 6 — ROC curves analysis to evaluate the discriminatory potential of mucosal microbial community combined with CCTC levels in Ducks’ stage identification. (A) Microbial community at the tumor mucosal classify Low- Ducks’ stage (stages 1–2) from high- Ducks’ stage (stages 3–4). (B) Microbial community combined with CCTC at the tumor mucosal classify Low- Ducks’ stage (stages 1–2) from high- Ducks’ stage (stages 3–4). (C) Microbial community at the adjacent normal mucosal classify low-Ducks’ stage (stages 1–2) from high-Ducks’ stage (stages 3–4). (D) Microbial community combined with CCTC at the adjacent normal mucosal classify low-Ducks’ stage (stages 1–2) from high-Ducks’ stage (stages 3–4). [file Image_6.jpg]
